# A Practical Curriculum Design and Learning Effectiveness Evaluation of Competence-Oriented Instruction Strategy Integration: A Case Study of Taiwan Skills-Based Senior High School

**DOI:** 10.3390/bs13010043

**Published:** 2023-01-04

**Authors:** Chin-Wen Liao, Ying-Ju Tseng, Yu-Hsiang Liao, Bo-Siang Chen, Wei-Sho Ho, I-Chi Wang, Hung-I Lin, I-Min Chen

**Affiliations:** 1 Department of Industrial Education and Technology, National Changhua University of Education Bao-Shan Campus, No. 2, Shi-Da Road, Changhua City 500208, Taiwan; 2 Center of Teacher Education, National Chung Hsing University, No. 145, Xingda Rd., South Dist., Taichung City 402202, Taiwan; 3 Department of Child Care and Education, National Yuanlin Home-Economics and Commercial Vocational Senior High School, No. 56, Zhongzheng Rd., Yuanlin City 510005, Taiwan; 4 Center of Teacher Education, Chaoyang University of Technology, No. 168, Jifeng E. Rd., Wufeng Dist., Taichung City 413310, Taiwan; 5 Department of Vehicle Engineering, Nan Kai University of Technology, No. 568, Zhongzheng Rd., Caotun Township 542020, Taiwan; 6 NCUE Alumni Association, National Changhua University of Education Jin-De Campus, No. 1, Jinde Rd., Changhua City 500207, Taiwan; 7 College of General Education, National Chin-Yi University of Technology, No. 57, Sec. 2, Zhongshan Rd., Taiping Dist., Taichung City 411030, Taiwan; 8 Department of Electrical Engineering, National Shiou Shuei Senior Industrial Vocational High School, No. 364, Zhongshan Rd., Xiushui Township 504008, Taiwan

**Keywords:** skills-based senior high school, strategies of competence-oriented instruction, learning engagement, learning effectiveness

## Abstract

The 12-Year Basic Education Curricula not only follow the objectives of previous curriculum syllabus development, but they place more focus on competence-oriented instruction, which aims to emphasize the importance of combining the curriculum with life situations that are not solely based on learning knowledge and skills. This study aims to investigate the results of the students’ learning effectiveness and learning engagement after adding competence-oriented instructional strategies into electrical engineering practical subjects offered by the Department of Electrical Engineering in skills-based senior high schools, and, at the same time, to figure out the difference in learning effectiveness using different instructional strategies. Two classes of students from the Department of Electrical Engineering major in electrical engineering practical subjects in one skills-based senior high school in Central Taiwan were chosen as the participants for this study. By way of pre-test–post-test research design and heterogeneous grouping, a 10-week instruction experiment consisting of two groups and occurring over the course of 30 classes was conducted, wherein competence-oriented instructional strategies were used in an experimental group, while traditional didactic instructional strategies were used in a control group. By analyzing the collection of quantitative and qualitative data through competence-oriented instructional strategies, the instruction effectiveness and feasibility of the basic electricity practical curriculum were developed as the study topic for understanding how competence-oriented instruction can be implemented into the practical curriculum of the electrical engineering and electronic engineering group. According to the research objective, the results were concluded as follows: (1) Students of the Department of Electrical Engineering have a slightly better learning effectiveness in electrical engineering practice under competence-oriented instructional strategies compared with those who learned under the traditional didactic instructional strategies; (2) there is a significant difference in the students’ learning engagement in electrical engineering practice from the Department of Electrical Engineering under competence-oriented instructional strategies compared with those who learned under the traditional didactic instructional strategies. The conclusion of this study emphasizes designing questions based on life situations, thereby applying what students have learned to solve problems they encounter in their daily lives. Compared with traditional didactic instructional strategies, competence-oriented instructional strategies not only have a better learning effectiveness and provide flexibility for the students to solve problems and provide analysis for situations, they also have broader applicability and an increased number of positive benefits when it comes to students’ group interactions and sharing.

## 1. Introduction

Traditional didactic instructional strategies are not able to adapt to the current period of globalization, and they fail to provide students with appropriate assistance to meet the needs of external industries, moreover, these strategies lack the ability to meet the expectations of globalization. The Organization for Economic Cooperation and Development (OECD) proposed the project, “The Future of Education and Skills 2030”, in 2018 to help countries of the world with educational innovations that focus on the competencies that students require and the appropriate instructional methods that teachers should use in order to effectively guide students to learn the attitudes and knowledge they need for the future [1].

The core competencies are the curriculum development targets for the 12-Year Basic Education Curricula, which aim to achieve the same results, i.e., cultivating students’ practical capabilities, as the previous curriculum’s guidelines do. Both the concepts of “literacy” and “ability” come from the English words “competence” and “competency”, in which competence means the internal capability of behavior performance, while competency stands for the internal quality and characteristics [2]. In 2018, the Ministry of Education emphasized that there is no fixed model for competence-oriented instruction, and that it is not the only standard. The competence-oriented instruction’s principles were developed from its meaning, and there are three dimensions and nine items of core competencies, i.e., interdisciplinary instruction key-points, and competence-oriented instruction has four cardinal principles (knowledge, skills, and attitude integration; situated learning; learning methods and strategies; utilization and implementation) for teachers to use to design curriculum [3]. The four key-points of competence-oriented instruction that were developed by Chan and Huang (2020) according to 108 new curriculum guidelines, sentiments, and domestic education scholars’ opinions are as follows: (1) To convert the learning key-points and form competence-oriented learning targets in knowledge, skills, and attitude; (2) to develop learning materials and connect them to real-world situational contexts in order to develop meaningful competence-oriented learning activities; (3) to guide students’ active investigation and implementation of what they have learned and, at the same time, to develop competence-oriented learning activities that help strengthen students’ capabilities to solve problems; (4) to plan and design situational practical tasks in order to encourage the integration and implementation of a competence-oriented learning process for students. In conclusion, when competence-oriented instruction implementation strategies are considered by teachers of different areas in which life-situated contexts are integrated into curriculum instruction, the students will be able to employ what they have learned in their daily lives, and at the same time, multi-level cognition and effective learning are developed, resulting in the development of the students’ self-directed learning, communication and interaction capabilities in learning activities and prompting engagement in social participation, and therefore, the students’ lifelong learning capabilities will be developed [4]. Tsai (2020) said that core competencies can be guided through education, which can be planned through the curriculum, cultivated through instruction, and evaluated through assessment, and the achievement can be gained through learning [5]. Chen et al.’s (2021) integrated a competence-oriented curriculum with field trips and used virtual reality experience tools to examine the students’ learning effectiveness, in which they found that the students would have acquired a multi-faceted understanding of expression, application, interpretation, and viewpoints regarding inquiry learning [6]. Deng (2018; 2022) developed a competence-oriented curriculum design for museums and schools, and also, they conducted team teaching in a competence-oriented curriculum through external resources and found that there are deep and broad learning effects in subjects of arts, nature, reading, and the comprehensive field curriculum [7,8]. Yu (2021) implemented a competence-oriented curriculum in as part of the project-based practical curriculum in a skills-based senior high school in which students in the Department of Control Engineering made good use of LEDs in the household environment, and he also figured out that when the curriculum was combined with reality, the teachers should help the students to produce the solutions and integrate the knowledge and skills to meet the requirement of the future [9]. As a consequence, the competence-oriented curriculum aims to promote teachers’ to positions of instructive and learning process guides and helpers, while the students are independent learners, and at the same time, this will encourage the students to connect their life situation context to their learning experiences in order to cultivate their capacity to integrate knowledge, skills, and affection, and also, to strengthen many of their interdisciplinary/cross-region and lifelong learning capabilities.

Electrical engineering and electronic engineering industries are Taiwan’s safeguarded industries. According to the statistics from the Ministry of Education in 2022, there were eighty-two thousand students majoring in electrical engineering and electronic engineering, which ranked at the top among the domestic universities and colleges in 2021, and business management and information technology ranked second. Among them, the number of students in the Department of Electrical Engineering, the Department of Electronic Engineering, and the Department of Business Management and Information Technology accounted for 18.2% of the total number of students [10]. In order to cultivate the grassroot, qualified technical personnel for the electrical engineering and electronic engineering related technology industries, students of electrical engineering and electronic engineering in skills-based senior high schools should be able to take charge of the operation, manufacturing, maintenance, testing, design, and work-related applications of electrical and electronic engineering, information technology, automatic control, refrigerating and air-conditioning, and communication networks, and meanwhile, their employability can be strengthened based on the core competencies that help students to improve their capacity to practice systematic thinking, the application of information technology, and coded identification so that they are able to systematically plan the circuit layout and solve circuit-related problems, which shows their cognitive abilities to perform information technology equipment applications, problem solving, communication and coordination, and teamwork competency [11]. Hung (2018) suggested that all the countries of the world contribute to the cultivation of talents and follow the world trends of horizontal integration in interdisciplinary/cross-region learning based on the subject knowledge, meanwhile, it is important to guide learners to investigate all the phenomena in their daily lives and to develop deep learning skills regarding innovation and connection [12]. According to Olympiou et al., in 2013, many types of demonstrations should be offered in the forms of figures, graphics, and symbols in order to strengthen the students’ concepts of learning and to enhance their knowledge understanding level [13]. As a consequence, nowadays, the top priority for connections-based learning is to apply a combination of images, animation, film, sound effects, characters, and audio multimedia techniques to the instruction curriculum. The research results from Lu (2021) showed that different topic curriculum arrangements and appropriate instruction activities appeal to the students’ learning interests, and meanwhile, there was a significant impact on the students’ learning attitude and curriculum satisfaction, in which their learning effectiveness was affected positively [14]. Yiu (2019) indicated that teachers should consider the students’ development and learning objectives when they are developing competence-oriented instructions, and they should plan different types of scaffolding theories, deepen the students’ learning by designing questions for them, add active investigation, conversation, practice, questioning, and proof activities into the learning process, and they also should guide the students to think, investigate, and understand so that they will grow during their learning [15]. In terms of the students of electrical engineering and electronic engineering, practical experiments on electrical circuits will allow the students’ to acquire basic knowledge and cultivate skills in applying new knowledge of electrical engineering, and hence, having a good or a bad curriculum design is of great concern in the students’ learning motivation and learning effectiveness. Therefore, it is a top priority for teachers to develop their instruction key points as they combine the competence-oriented instructional strategies and the curriculum’s instructional materials and as they design leading informational and real life-situated learning materials in order to make the curriculum more practical and industrial, and by doing this, they may follow the future trends, in which the students’ learning effectiveness could be improved.

## 2. Literature Review

### 2.1. Electrical Engineering and Electronic Engineering Group in Skills-Based Senior High School

In this study, the conceptual definition of a “Skills-based Senior High School” means that the main curriculum designed to provide professional and practical subjects, including a practical skill program and cooperative education [16]. According to the curriculum guidelines in electrical engineering and electronic engineering, of which there are 108 curriculum guidelines, the development goal in the curriculum is to cultivate the students’ basic skills for their future work. Providing an interdisciplinary/cross-region curriculum design for electrical engineering and electronic engineering emphasizes the importance of core competencies in the study area, in which students are able to obtain the basic level of competency relevant to the job requirements and to adapt to the rapid changes in the workforce in the future. Studying electrical engineering and electronic engineering cultivates the students’ knowledge and practical skills in electrical and electronic engineering, information technology, automatic control, refrigerating and air-conditioning appliances, and the communication network, and at the same time, this integrates what the students have learned with the industry development trends so that the curriculum’s development keeps in line with that of the industry techniques, and it also strengthens the students’ skills and service attitude [11].

The definition of operational requirement relates to the students who participate in the practice of electrical engineering and electronic engineering in this study. The curriculum focuses on the cultivation of the students’ basic knowledge in electricity, and it also improves their circuit assembly, analysis, design, and application skills by following the core competencies in electrical engineering and electronic engineering, which are considered to be the foundation stone for students learning electrical engineering and electronic engineering, and these help to build their creative thinking, systematic thinking, and problem-solving capabilities. The curriculum of professional practice mentioned above is a compulsory requirement by the Ministry of Education, and consequently, it was chosen as the research area to be evaluated for this study.

### 2.2. Strategies of Competence-Oriented Instruction

The competence-oriented instructional strategies of Taiwan were derived from the grade 1–9 curriculum, which was officially implemented in 2002. The grade 1–9 curriculum is the curriculum that is applied in elementary school (grade 1–6) and junior high school (grade 7–9), and the grade 1–9 curriculum emphasizes the targets which cultivate the students’ capabilities in 10 core competency areas through competence indicators or benchmarks, including self-understanding and potential development; appreciation, performance, and innovation; career planning and lifelong learning; expression, communication, and sharing; respect, care, and teamwork; cultural learning and international understanding; planning, organizing and implementing; the utilization of technology and information; active investigation and research; independent thinking and problem solving [17]. Yang, Li, Zhang, and Zhang (1999) considered the competence indicators or benchmarks as the transformation of the competence items that the students should possess into factual data for the instructors to observe and evaluate, from which the students’ learning performance is reflected [18]. In Chou’s research in 2003, there were more than 20 items, with 2 to 3 items in each section in the 10 core competencies in Taiwan, and unlike in countries such as Australia, the United Kingdom, and the USA, the core competence is simple and fundamental [19]. Since 2013, the Taiwan government announced its plan to promote the 12-Year Basic Education Curricula based on the success of the launch of grade 1–9 curriculum guidelines, and the 12-Year Basic Education Curricula was implemented until 2018, with the aims to cultivate children’s practical capabilities, problem-solving capabilities, and life-long learning capabilities. There is a large similarity with these ones and the key competencies in the European Reference Framework proposed in European Union (EU) in 2018. These key competencies include literacy competence; multilingual competence; mathematical competence and competence in science, technology, and engineering; digital competence; personal, social, and learning competence; citizenship competence; entrepreneurship competence; key competencies for lifelong learning; cultural awareness and expression competence [20]. The Core Competencies in Taiwan refer to the Education 2030 Learning Framework, which was proposed by OECD in 2018, in which the competencies are considered to be part of the students’ learning portfolio, taking root in their personal knowledge, skills, attitude, and values, which will eventually enter the core central area of the concentric zone model and help them to achieve the ultimate goal of the personal and social wellness in society. The Core Competencies in Taiwan cover the concepts of both competency and literacy, in which one’s knowledge, skills, and attitudes that they gained after being educated would be enable them to proactively react and respond to the comprehensive situation and status of the personal and social demands of life at the present moment and in the future, and eventually, they could achieve the core goal of becoming a lifelong learner based on the basic ideas of spontaneity, communication, and interactions and social participation in the concentric zone model. Moreover, with a corresponding basic idea in the curriculum guidelines, the learners’ autonomous actions work in the concentric zone model during the real-life situation of the cultivation of a future civilization [1,21].

The National Academy for Educational Research defined the “Core Competencies” as the knowledge, skills, and attitude of one person who has to adapt to their life and future challenges, in which the subjectivity of the learners’ learning should be manifested, rather than seeing the content knowledge as the only category [22]. Taiwan PISA National Education Research Center defines “Core Competencies “ as “the literacy and ability that students should have when facing various situations and challenges in daily life” [23]. Tsai (2020; 2021) indicated that “Competence oriented” means a type of curriculum and instruction base that has a combination of “Subject-Specific Competencies” and “Core Competencies” [24,25]. Chao (2018; 2019; 2021) suggested that teachers should focus on the students’ learning process when they are conducting the competence-oriented strategies in order to more accurately understand their learning effectiveness, in which cognition, skills, and affection cannot be neglected. Teachers should appropriately integrate multiple ways into the learning assessments [26,27,28]. In this study, the conceptual definition of “Strategies of Competence-oriented Instruction” emphasize the connection between the situated context during the instruction process, the students’ active participation, and the integration of care cognition, knowledge, skills, and affection [3].

The definition of operational requirement is considered, in this study, as the competence-oriented instructional strategies in electrical engineering practical subjects in the Department of Electrical Engineering, which have been developed based on the core competencies of the course syllabus. In terms of the situated instruction activities in the competence-oriented curriculum, the teachers could design the activities (preparing stage) by setting situated tasks as the starting point, and then the students’ learning motivation would be encouraged because these concern the problems they encounter in their daily lives, and they will be need to solve the problems. During in developing stage, the teachers could design the instruction activity materials around to life situation and give hints for the situated tasks to guide students to solve the problems and complete the tasks by making good use of the strategic knowledge they have acquired in of the process of implementing their learning. In addition, the situated tasks could be designed to include incident descriptions about a incident, situation, story, and context that last from 100 to 250 characters [29].

### 2.3. Learning Engagement

Fredricks et al. (2004) thought that learning engagement is the key factor to achieve effective student learning, in which the students’ learning effectiveness scores can be evaluated through their learning engagement in the short term, while the students’ memory and learning effectiveness degree can be evaluated in the long term [30]. In Kuh’s opinion (2009), learning engagement is the performance of knowledge, skills, and attitude by learners, in which the time and effort the learners apply are considered to be the indicators of it, and by communicating with others, the students’ learning process could be positively developed. In this study, the conceptual definition of “Learning Engagement” stands for the performance of knowledge, skills, and attitude between learners and their learning activities process [31]. Furthermore, according to the point of view of Fredricks et al. (2004), learning engagement is divided in to two components, emotional engagement and cognitive engagement [30].

The definition of operational requirement is considered, in this study, to be the scores on the learning engagement scales of the participants, in which the higher scores reflect a deep level of learning engagement, while, the lower scores reflect a more shallow level of learning engagement.

### 2.4. Learning Effectiveness

Yin (2018) indicated that learning effectiveness means the degree of understanding of the issues in the curriculum, the students’ learning satisfaction, and the qualitative achieved by the learners [32]. Lai (2013) thought that learning effectiveness is the learners’ performance and achievement after a certain period of time, which can be measured using every type of testing or assessment tool that has been designed or made according to the instruction objectives [33]. Peng and Ho (2013) indicated that the knowledge and skills that the students gained by performing the school’s learning activities could be proven through their performance in the learning process and their assessment scores [34]. In this study, the conceptual definition of “Learning Effectiveness” stands for the transformation in the knowledge, skills, and attitude that the learners gained during their learning process after completing the instruction activities that were set by the teachers [35]. Furthermore, according to the point of view of Yu and Han (2009) and Chen and Chang (2019), “Learning Effectiveness” is divided in to two components, the performance in paper and pencil tests and in simulated performance tests [36,37].

The definition of operational requirement is considered, in this study, to be the scores in the performance evaluation of the participants. The evaluation content includes paper and pencil testing and simulated performance testing, in which the scores were calculated by the use of performance evaluation scoring rubrics. Lin (2020) indicated that learning effectiveness evaluation includes learning, behaviors, and achievement, which help the students to increase their learning requirement, learning interests, learning engagement, and learning effectiveness scores [38]. The performance evaluation scoring rubrics are divided into knowledge, skills, and attitude (three components and five levels) with the performance scores having a range of 20 points. The sum of the figures in three components in the performance evaluation scales is the total score. The higher the score is, the deeper the learner’s learning engagement is, while the lower the scores is, the more superficial the learner’s learning effectiveness is.

## 3. Methodology

### 3.1. Research Design

This study uses a quasi-experimental design. The instruction environment was controlled before the instruction experiment was conducted, in which the control variables were the student grades, the instructors, the number of instruction hours, the students’ degree, the instruction content, and the evaluation content. This study aims to investigate the relationship of the students’ learning effectiveness and learning engagement in electrical engineering practical subjects between competence-oriented instructional strategies and the traditional didactic instructional strategies. The differences are shown as follows:

1. Experimental group: competence-oriented instructional strategies. The implementation of a horizontal connection between the instruction activities and instructional materials helps the students to develop of an understanding of a concrete concept, observe their daily life experiences, and gather information about industry developments, address and analyze a situation, investigate a problem, and perform interdisciplinary/cross-region thinking.
2. Control group: traditional didactic instructional strategies involve the implementation of didactic instructions and operation demonstrations by the teachers according to the content and the schedule of the textbook.

**Figure 1:**
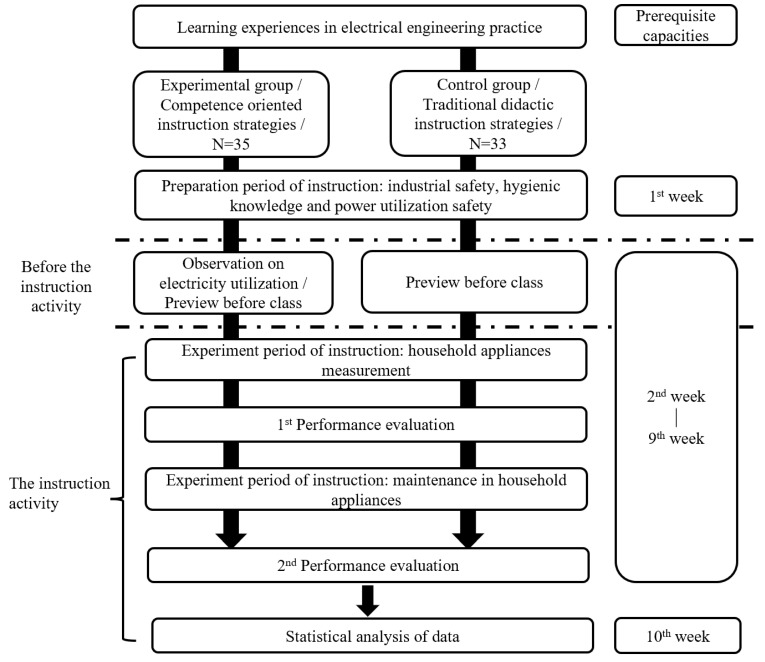
Estimation of path parameters of mediation effect model.

The instruction experimental design is shown in Table 1, and the instruction experiment progress is shown in Figure 1.

### 3.2. Participants

Two classes of students who are Department of Electrical Engineering majors in electrical engineering practical subjects in one skills-based senior high school in Central Taiwan were chosen as the participants for this study. Those students completed the electrical engineering practice curriculum before the instruction experiment began. There were a total of 35 students in the experimental group, in which the competence-oriented instruction strategy was conducted, and there were total 33 students in the control group, in which the traditional didactic instruction strategy was conducted. The instructor was the original instructor in the Department of Electrical Engineering of both of the classes, and he graduated from the same department and has a large amount of teaching experience. The instruction strategy was the only difference between the two classes, while the learning content and hours were the same. The competency assessments were conducted by the participants in this study before the instruction experiment, and the students were ranked according to their assessment scores, in which the top fifty one them were placed into a high-score group, while the other fifty of them were placed into a low-score group. Then, one student in the high-score group and one student in low-score group were chosen as the members of the cooperative learning group. Both the classes adopted the heterogeneous and two-part grouping based on the competency assessment results.

### 3.3. Instruction Experiment Progress

The instruction experiment progress was conducted for 10 weeks (for three hours each week), and there were a total 30 instruction hours including the preparation week and the final examination week. Before the instruction experiment, the students in both the experimental group and the control group accepted the competency assessments which were considered to be means of evaluating the basis of the students’ prerequisite capacities and the heterogeneous grouping. During the instruction experiment period, there were two performance evaluations, which were designed according to the unit implementation content, in which the first evaluation was mostly made of choice questions and essay questions concerning the students’ learning background, while the second evaluation took the form of multiple assessments, in which students solved problems using the knowledge and skills they had learned in class, and at the same time, open-ended questions were added into the second evaluation. A collection of the qualitative data, the instruction process, and the curriculum distribution are shown as Figure 1.

### 3.4. Instructional Strategies Progress

**Figure 2:**
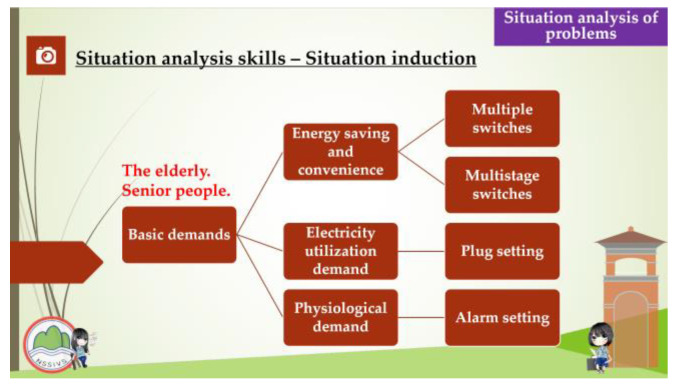
Competency-based instructional materials used in this research. These require situation analysis skills.

**Figure 3:**
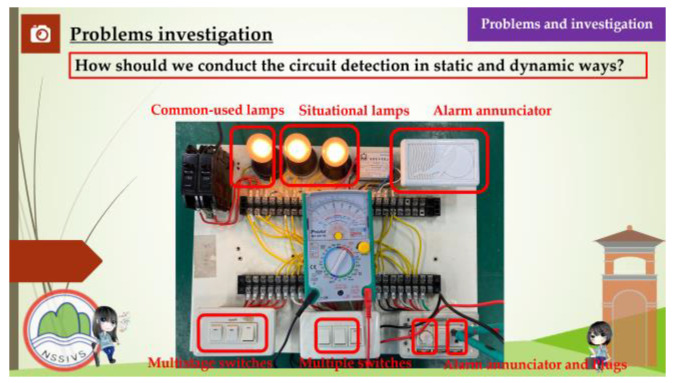
Competency-based instructional materials used in this research. This is the problem investigation.

**Figure 4:**
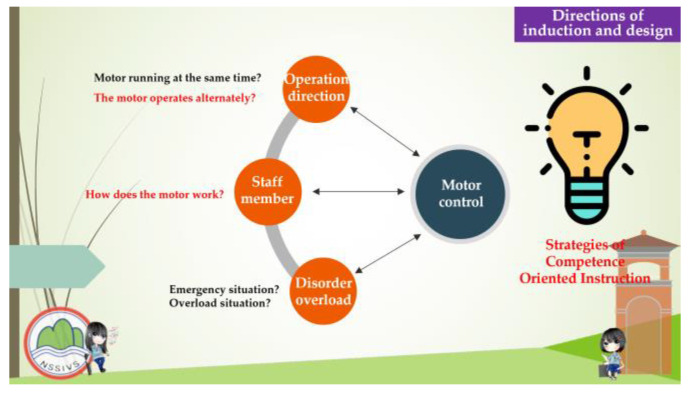
Competency-based instructional materials used in this research. These involve the ideas of induction and design.

**Figure 5:**
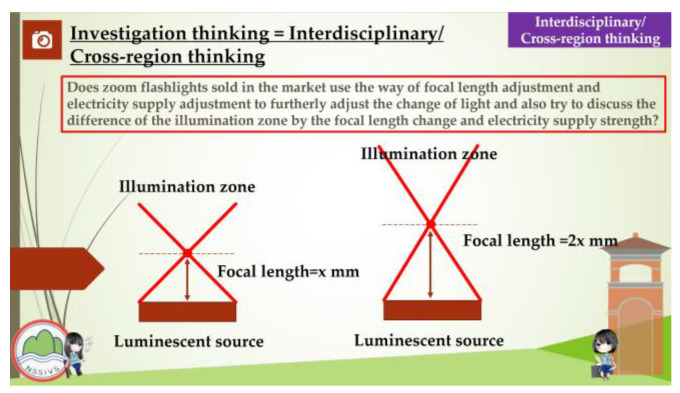
Competency-based instructional materials used in this research. These require interdisciplinary/cross-region thinking.

This study aimed to investigate the relationship between the competence-oriented instructional strategies and the traditional didactic instructional strategies which were used to assess the students’ learning effectiveness and learning engagement in electrical engineering practical subjects. The students of experimental group recalled and organized the experiences that occurred in their daily lives and related them to the instruction curriculum before the instruction experiment was performed, and the same time, they shared their opinions during the instruction, while the students of the control group learned according to the traditional didactic instructions that were given by the teachers. The instruction content focused mainly on the following four units: “Way to Use The Basic Electrical Tools”, “Assembly of The Switch, Plug, and Appliances”, “ Electric Motor Reversible Control” and “Electric Motor Cycle Control”. The competence-oriented instructional strategies emphasize the implementation of the horizontal connection between the instruction activities design and the instructional materials, in which the instructors helped the students to develop an understanding of a concrete concept, observe their daily life experiences, and gather information about industry developments after receiving instructions about the circuit application, energy education, and safety education-related issues. Concerning the factors such as the number of instruction hours the depth of the issue integration, and the students’ capabilities development, the activities, the part of the curriculum content, and the related data were organized during the instruction period to help the students to address and analyze a situation, investigate a problem, and perform interdisciplinary/cross-region thinking. The competence-oriented instructional materials are shown in Figure 2, Figure 3, Figure 4 and Figure 5, as follows.

**Figure 6:**
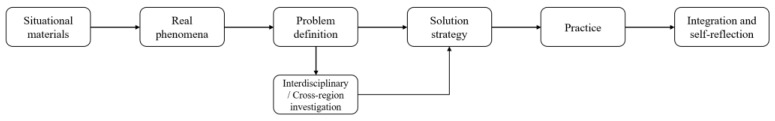
Process flow of competence-oriented instruction.

The competency-based instructional process is shown in Figure 6, with brief descriptions, as follows:

1. Situational materials: these are used to guide the students to identify the types of household appliances, the methods that could be used, and the fields of utilization for household appliances in their daily lives and to try to figure out the difference between the utilization and planning of the appliances.
2. Real phenomena: these are applied to allow the students to observe the results in a situational context and also to generalize and analyze the phenomena, such as the relationship between the automatic door switches and the electric motor. Are there any alternatives?
3. Problem definition: this is used to define the scope and to distinguish the problem area according to the problems of real phenomena. For example, the automatic door sensors are too sensitive, which will cause the abnormal launch of the doors. What is the solution to the problem?
4. Interdisciplinary/cross-region investigation: this allows the students to find related instructional materials and to investigate the interdisciplinary/cross-region problems. For example, the automatic door sensors detect based on the phototonus method. What are the diffraction conditions of the light?
5. Solutions: these allow the students to implement the planning of a circuit design, static testing, dynamic testing according to the content of the “problem definition”.
6. Operation practice: this allows the students to implement the practical operation according to the circuit design content, static testing, and dynamic testing planning.
7. Integration and self-reflection: these allow the students to produce a generalization and an analogy of the problems encountered in the circuit design stage and in the practical process to avoid repeating mistakes.

**Figure 7:**
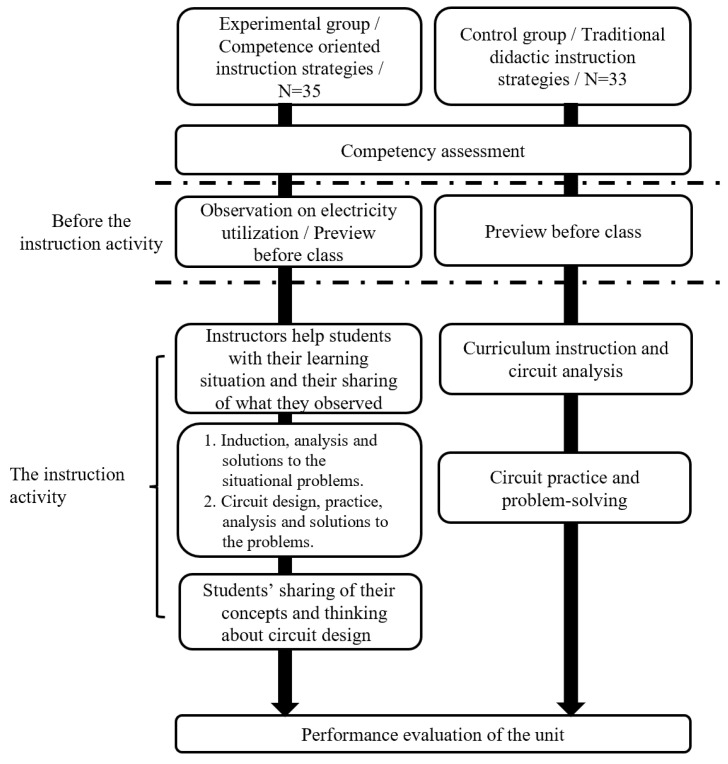
Process flow of experimental group and control group unit instruction implementation.

Concretely speaking, the unit content of both the groups were the same. Only the instructional materials and guiding methods used in the preview before the class and during the instruction process were different, while the other instruction steps were the same. However, the major difference is the impact of the series of the instructional model on the students’ learning process, not only the difference in the curriculum explanation. The students in the experimental group defined the problems, performed interdisciplinary/cross-region thinking, solved the problems by using teamwork strategies, generalized, and conducted generalization and self-reflection by means of combining the life material guiding with the practical situated problems guiding. There was a significant difference between the problem-solving and thinking processes that they encountered in building stage and those of the control group. Therefore, the major difference between the two groups should be considered as the difference between the competence-oriented instructional strategies and the traditional didactic instructional strategies, as shown in Figure 7 below.

### 3.5. Research Hypotheses

**H1.**  *Competence-oriented instructional strategies affect the learning effectiveness of electrical engineering practice learners.*

**H1-1.**  *Competence-oriented instructional strategies have positive effects on the learning effectiveness of electrical engineering practice learners in terms of the knowledge component.*

**H1-2.**  *Competence-oriented instructional strategies have positive effects on the learning effectiveness of electrical engineering practice learners in terms of the skill component*.

**H1-3.**  *Competence-oriented instructional strategies have positive effects on the learning effectiveness of electrical engineering practice learners in terms of the attitude component.*

**H2.**  *Competence-oriented instructional strategies affect the learning engagement of electrical engineering practice learners.*

**H2-1.**  *Competence-oriented instructional strategies have positive effects on the learning engagement of electrical engineering practice learners in terms of the emotional engagement component.*

**H2-2.**  *Competence-oriented instructional strategies have positive effects on the learning engagement of electrical engineering practice learners in terms of the cognitive engagement component.*

### 3.6. Research Tools

The following scales, including the (1) competency assessment scale, (2) the learning engagement scale, and (3) the performance evaluation scale, were used in this study. The competency assessments were conducted in both the experimental group and the control group by the students in order to figure out the participants’ initial behavioral learning experiences before the instruction experiment. After the instruction experiment, the participants were post-tested using the “learning engagement scale” and the “performance evaluation scale” in order to understand the difference between the periods before and after the experiment instruction regarding the participants’ learning effectiveness. The “performance evaluation scale” was used twice after the completion of the periodic curriculum.

#### 3.6.1. Competency Assessment Scale

The “competency assessment scale” was developed using the instructional materials of a common course syllabus in a junior high school, and it was divided into three components of knowledge, skills, and attitude, which were used as testing objectives according to the assessment feature, in which 150 valid samples on the competency assessment scale were collected. Kuo (2022) indicated that the higher the norm-referenced assessment questions discrimination degree is, then the higher the assessment’s reliability will be, in which the accepted minimum standard is over 0.25, and the standard difficulty index is 0.40–0.80 [39]. The test questions items were adjusted and decreased from 20 to 10 in accordance with the discrimination, which allowed it to meet the discrimination standard of greater than or equal to 0.25, while the difficulty standard was 0.40–0.80.

#### 3.6.2. Learning Engagement Scale

The learning engagement scale used in this study referred to the “behavioral learning engagement scale”, the “emotional learning engagement scale”, and the “cognitive learning engagement scale” developed by Chang, Lin, and Chou (2012), and it was modified into the version of the learning engagement scale which met the requirement of this study [40]. The original reliability value of the emotional learning engagement scale is 0.803 in Cronbach’s α, while the value of the cognitive learning engagement scale is 0.850. In terms of emotional learning engagement, there were six questions, which were designed to fit Likert’s five-point scale, in which the question items were divided into positive statement test questions and negative statement test questions. In the positive statements, the participants achieved 1 point for a response of “Strongly Disagree”; 2 points for “Disagree”; 3 points for “Neither Agree or Disagree”; 4 points for “Agree”; 5 points for “Strongly Agree”, while the negative statements were scored the opposite way. In terms of cognitive learning engagement, there were five questions, which were designed to fit Likert’s five-point scale, in which the participants obtained 1 point for the response of “Never”; 2 points for “Rarely”; 3 points for “Sometimes”; 4 points for “Often”; 5 points for “Always”.

#### 3.6.3. Performance Evaluation Scale

**Figure 8:**
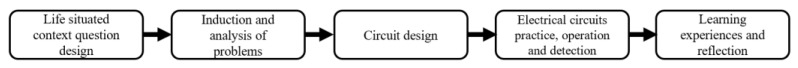
Process flow of situational performance evaluation.

The “performance evaluation scale” was developed using the point of view of Yu and Han (2009), Chen and Chang (2019), and Lin and Chen (2019) about the practical core competency assessment, and we also considered both the instructional materials produced by the National Academy for Educational Research and the characteristics of the practical curriculum in this study [36,37,41]. There were four question items in the assessment, and the first performance evaluation mainly relied on paper and pencil tests, and these were supplemented by a performance evaluation concerning of the participants’ learning background. The second performance evaluation was conducted in the form of many situated assessments, in which the situational statements were added. The students analyzed, organized, and simulated based on the situated context and the knowledge and skills they had learned in class, then, they designed the circuit according to their inductive analysis and the results of the situated content and used the circuit design to simulate practice, operation, and detection. Eventually, they were required to write down their learning experiences and reflect on them. The evaluation process is shown in Figure 8, as follows.

The performance evaluation rubric scale was classified into three components: knowledge, skills, and attitude, using the performance evaluation scoring viewpoint of Brookhart (2013), Chen (2017), Tang (2019), and Kelly (2019), and it was developed by adopting the rating scale of rubrics [42,43,44,45]. The evaluation items were divided into 3 parts: knowledge (K), skills (S), and attitude (A), and 5 performance levels in which the critical points of the evaluation were stated and listed; knowledge (K) occupied 34%, skills (S) occupied 33%, and attitude (A) occupied 33% of the total scores in the learning performance part. There were 20 points in the performance range over the five performance levels. The performance evaluation was conducted by 3 observers (1 instructor and 2 lesson-observing teachers), and the scoring was conducted by them, in which the average value was considered to be the practical evaluation grades of the students. The evaluation rubric scale developed in this study is shown in Table 2, as follows.

## 4. Data Processing and Analysis

In this study, the independent samples *t*-test was used to examine the difference between students in both the control group and the experimental group regarding their learning effectiveness in basic electricity practical subjects. The academic performance in electrical engineering practice was considered to be the covariance that was used to conduct the homogeneity test of overall learning effectiveness, and a further investigation was conducted to see if the students’ learning effectiveness scores would differ according to the implementation of different instructional strategies under the condition of excluding the impact of the students’ prior knowledge. Meanwhile, the opinions and feedback from the three observers during the instruction and the results of the two performance evaluations were induced and analyzed, supported by the qualitative data in order to conduct the instruction activities, time distribution adjustment, and the triangulation of the qualitative data.

### 4.1. Competence-Oriented Scale

Competency assessments were conducted in both the experimental group and the control group students in order to figure out the participants’ initial behavioral learning experiences before the instruction experiment. The data were evaluated using the independent samples *t*-test to examine the difference between the two groups by the independent samples.

### 4.2. Learning Engagement Scale

To exclude the impact of the competency assessment scores, an analysis of covariance was conducted in this study to investigate whether the students’ learning effectiveness in electrical engineering practical subjects would be affected according to the implementation of different instructional strategies. The competency assessment results were considered to be the covariance, the instructional strategies were considered to be the independent variable, and the overall learning effectiveness (the average value of the first and the second performance evaluations) was considered to be the dependent variable.

### 4.3. Performance Evaluation

Open-ended questions were designed in the performance evaluation in order to figure out the problems that the students encountered in the practice process, the solutions to the problems, and the students’ learning experiences and reflection, and at the same time, this was conducted to understand the effectiveness of the implementation of the research. The collection of the qualitative data was performed in the second performance evaluation, with there being 35 testers each time (twice) and 70 copies in total. The qualitative data were encoded according to the research objectives, and the encoding content contained the three components: knowledge, skills, and attitude. The encoding content and the definition were formed based on the performance evaluation rubric of electrical engineering practice in this study. After the performance evaluation, the analysis using the open-ended questionnaire by the participants was conducted by the researchers first. Then, after the discussion with the advisors about the appropriateness of the data analysis, the analyzed results were passed to the common analyzers (one instructor and two lesson-observing teachers) in order to figure out the problems that the students encountered in the practice process, the solutions to the problems, and the students’ learning experiences and reflection, and at the same time, they aimed to understand the effectiveness of the implementation of the research. The collection of the qualitative data was conducted in the first week of the instruction experiment period, in which the feedback from both the tested students and the two lesson-observing teachers was encoded. The analyzed results were subject to a lot of negotiations by the common analyzers until all the encoding decisions reached a consensus.

The research of the qualitative data implemented in the instruction strategy refer to the analytic method for analyzing qualitative data by Chen (2010 and 2020), who adopted triangulation as a data checking method [46,47]. The resource for the qualitative data analysis was mostly the feedback from the tested students and the lesson-observing teachers. The tested students and the lesson-observing teachers were required to finish the open feedback form after each class, in which the tested students could hand in the form of their own will. The problems that the students encountered in the practice process, the solutions to the problems, and the students’ learning experiences and reflections were understood from the feedback they included in the form.

## 5. Results and Discussions

### 5.1. Research Hypothesis and Empirical Evidence

#### 5.1.1. Prior Knowledge Analysis

According to Table 3, the “Recapitulation sheet of students’ prior knowledge scores”, when the students participated in the electrical engineering practice curriculum before the instruction experiment, the scores of the control group were on average 0.4 higher than they were in the experimental group. After the academic performance was conducted in the independent samples t-test, the t value was 0.018, *p* = 0.985 > 0.05, which is below the level of significance. The result shows that there is no significant difference in the background capabilities of the students before they participated in basic electricity practical subjects among both the groups.

Further, from Table 4, the “Recapitulation sheet of students’ prior knowledge scores in knowledge, skills, and attitude”, the average score of the control group students was 1.78, which is slightly lower than that of the experimental group in terms of the knowledge component, and the t value was −2.351, *p* = 0.022 < 0.05, which reaches the level of significance; the average score of the control group students was 1.85, which is slightly higher than it is in the experimental group in terms of the skill component, and the t value was 2.610, *p* = 0.011 < 0.05, which reaches the level of significance; the average score of the control group students was 0.04, which is slightly lower than that of the experimental group in terms of the attitude component, and the t value was −0.043, *p* = 0.966 > 0.05, which fails to reach the level of significance.

#### 5.1.2. Variance Analysis in Basic Electricity Practical Subjects under Different Instructional Strategies

The learning effectiveness assessment results in each stage are shown in Table 5, the “Recapitulation sheet of basic electricity practice learning effectiveness scores”, with a brief description as follows:

The first assessment of learning effectiveness: The assessment was conducted in the 5th week of the instruction experiment, and the testing content included measurement and application in a household electricity utilization environment. The test results show that the average score of the experimental group students was 1.2 higher than that of the control group, and the t value was −0.472, *p* = 0.639 > 0.05, which fails to reach the level of significance. From the further comparison of the students’ assessment performance in the first assessment of learning effectiveness between the two groups in knowledge, skills, and attitude from Table 6, Table 7 and Table 8, the test results show that the average score of the experimental group students in terms of the knowledge component was 0.31 higher than that of the control group, and the t value was −0.323, *p* = 0.748 > 0.05, which fails to reach the level of significance; the average score of the experimental group students in terms of the skill component was 0.6 higher than that of the control group, and the t value was −0.649, *p* = 0.519 > 0.05, which fails to reach the level of significance; the average score of the experimental group students in terms of the attitude component was 0.29 higher than that of the control group, and the t value was −0.434, *p* = 0.666 > 0.05, which fails to reach the level of significance.

The second assessment of learning effectiveness: The assessment was conducted in the 9th week of instruction experiment, and the testing content included measurement and application in a household electricity utilization environment. The test results show that the average score of the experimental group students was 6.08 higher than that of the control group, and the t value was −2.928, *p* = 0.005 < 0.05, which reaches the level of significance. From the further comparison of the students’ assessment performances in the second assessment of learning effectiveness between the two groups in knowledge, skills, and attitude from Table 6, Table 7 and Table 8, the test results show that the average score of the experimental group students in terms of the knowledge component was 1.93 higher than that of the control group, and the t value was−2.823, *p* = 0.006 < 0.05, which reaches the level of significance; the average score of the experimental group students in terms of the skill component was 2.51 higher than that of the control group, and the t value was −3.131, *p* = 0.003 < 0.05, which reaches the level of significance; the average score of the experimental group students in terms of the attitude component was 1.64 higher than that of the control group, and the t value was −2.536, *p* = 0.014 < 0.05, which reaches the level of significance.

#### 5.1.3. Variance Analysis in Basic Electricity Practical Subjects under Different Instructional Strategies

Analysis of the overall learning effectiveness:

(1) Homogeneity of regression coefficients within the groups in the overall learning effectiveness data.

The homogeneity test of the regression coefficient in the overall learning effectiveness data is shown in Table 9, in which F = 2.192, *p* =0.144 > 0.05 (which is below the level of significance), and therefore, the one-way analysis of covariance (One-way ANCOVA) could be conducted in a later step.

(2) Analysis of covariance (one-way ANCOVA) in overall learning effectiveness.

Excluding the impact of the prior knowledge both the groups on the students’ overall learning effectiveness, the testing result was F = 7.239, *p* = 0.009 < 0.05, which reaches significant difference, and this means that the overall learning effectiveness in electrical engineering practice was affected according to the instructional strategies shown in Table 10. From the recapitulation sheet of statistics in relation to the overall learning effectiveness data from the basic electricity practical (Table 11), the adjusted average scores of the experimental group students were 3.67 higher than those of the control group, and the average score of the experimental group students was 6.08 higher than that of the control group, which shows that the integration of competence-oriented instruction into the basic electricity practical has a positive effect on the overall learning effectiveness.

Analysis of overall learning effectiveness in knowledge component:

(1) Homogeneity test of the regression coefficient from the overall learning effectiveness data in terms of the knowledge component.

In the homogeneity test of the regression coefficient from the overall learning effectiveness data in terms of the knowledge component (Table 12), the value was F = 2.725, *p* = 0.104 > 0.05 (which is below the level of significance), and therefore, the analysis of covariance (one-way ANCOVA) could be conducted in a later step.

(2) Analysis of covariance (one-way ANCOVA) in the overall learning effectiveness in terms of the knowledge component.

Excluding the impact of prior knowledge in both the groups on the students’ overall learning effectiveness, the testing result was F = 0.002, *p* =0.966 > 0.05, which fails to reach significant difference, and this means that the overall learning effectiveness in the electrical engineering practical was be affected according to the instructional strategies shown in Table 13. From the recapitulation sheet of statistics on the overall learning effectiveness of the basic electricity practical in terms of the knowledge component (Table 14), the adjusted average score of the experimental group students was 0.003 higher than that of the control group, which shows that the integration scores of the competence-oriented instructions in terms of the knowledge component with the basic electricity practical are below the level of significance in statistics, nevertheless, the average score in overall learning effectiveness of the experimental group was higher than that of the control group.

Analysis of overall learning effectiveness in skill component.

(1) Homogeneity of regression coefficients within the groups in overall learning effectiveness in terms of the skill component.

In the homogeneity test of the regression coefficient in relation to the overall learning effectiveness in terms of the skill component (Table 15), the value was F = 0.366, *p* = 0.547 > 0.05 (which is below the level of significance), and therefore, the analysis of covariance (one-way ANCOVA) could be conducted in a later step.

(2) Analysis of covariance (one-way ANCOVA) in relation to overall learning effectiveness in terms of the skill component.

Excluding the impact of prior knowledge in both the groups on the students’ learning effectiveness in terms of the skill component, the testing result was F = 28.983, *p* = 0.000 < 0.05, which is a significant difference, and this means that the learning effectiveness in terms of the skill component of electrical engineering practice was affected according to the instructional strategies shown in Table 16. From the recapitulation sheet of statistics in the overall learning effectiveness of the basic electricity practical in terms of the skill component (Table 17), the adjusted average score of the experimental group students was 2.96 higher than that of the control group, which shows that there is positive impact of the integration of competence-oriented instructions on the students’ overall learning effectiveness in the basic electricity practical.

Analysis of overall learning effectiveness in attitude component.

(1) Homogeneity test of regression coefficient in relation to overall learning effectiveness in terms of the attitude component.

In the homogeneity test of the regression coefficient in relation to the overall learning effectiveness in terms of the attitude component (Table 18), the value was F = 0.008, *p* = 0.931 > 0.05 (below the level of significance), and therefore, the analysis of covariance (one-way ANCOVA) could be conducted in a later step.

(2) Analysis of covariance (one-way ANCOVA) in overall learning effectiveness in terms of the attitude component.

Excluding the impact of prior knowledge in both the groups on the students’ learning effectiveness in terms of the attitude component, the testing result was F = 5.227, *p* = 0.026 < 0.05, which is a significant difference, and this means that the learning effectiveness in terms of the attitude component of electrical engineering practice was affected according to the instructional strategies shown in Table 19. From the recapitulation sheet of statistics in the overall learning effectiveness of the basic electricity practical in terms of the attitude component (Table 20), the adjusted average score of the experimental group students was 0.95 higher than that of the control group, which shows that there is positive impact of the integration of competence-oriented instructions on the students’ overall learning effectiveness in the basic electricity practical.

#### 5.1.4. Inductive Analysis of Qualitative Data in Instructional Strategies Implementation

During the instruction experiment period, the opinions and feedback from the three observers and the results of the two performance evaluation scores were used as the references for the instructional activities and time adjustment, including the situated instruction, interdisciplinary/cross-region learning, graphics reading, and practice time distribution, etc. The qualitative data of the instruction strategy in this study were their feedback, which was collected from two instructors and the students in the experimental group after the instruction experiment, and in total, there we obtained 24 copies in 8 weeks. After the triangulation of the qualitative data, which were collected from the instructor, one lesson-observing teacher, and the students, the results that are shown in Table 21, Table 22 and Table 23 were obtained.

The results of Table 21 (a statistical table of a collection of organized, qualitative data including positive feedback and common opinions that were gathered during the competence-oriented instructional strategy implementation) show that the top three items in the sequence, which the three observers focused on, were “situational connection and expression-positive”, “reading ability”, and “integrate interdisciplinary/cross-region and issues into subjects”.

As a part of “situational connection and expression—positive”, the coefficient of agreement was 0.83, which means that teachers and students positively confirmed the connection between the instruction content and the situated context. The situated connection helps the students to deepen their knowledge and capabilities, and at the same time, this reduce the discrepancy between learning and using. The lesson-observing teachers suggested that the integration of the situated context might be conducted by observing the daily events during life and in industries, and also by the guiding the curriculum’s instruction design in order to make the students learn completely from the knowledge they were able apply to their lives. In addition, internet multimedia groups are also a good way to help students with their learning and discussion.

As a part of “reading ability”, the coefficient of disagreement was 0.67. From the instruction activities and the situated description, the lesson-observing teachers found that there was an application deficiency in the students in alphabet and graphics reading. For example, the students were not able to integrate the situated requirement and figure information during the instruction activities, which caused the discrepancy in cognitive expression. In terms of the cultivation of the “reading ability”, the suggestion from the lesson-observing teachers involved questioning them first, and then guiding their investigation and comprehension and problem definition in textual or situational reading and related fields.

As a part of “integrate interdisciplinary/cross-region and issues into subjects”, the coefficient of agreement was 0.58. The students in the experimental group used the interdisciplinary/cross-region means to answer the question on the selection of common lamps and environment light requirements. The design helped the students to understand lamp applications and investigate optical imaging through optical glass adjustment.

As stated above, the results of this study show that the competence-oriented instructional strategies had positive effect on the students’ overall learning effectiveness, in which there is significant difference in the skills and attitude components between the two groups. The following text includes the discussion about the evaluation of each stage.

After the overall evaluation of the two learning effectiveness performances and in reference to the qualitative data including common opinions of Table 21, “a statistical table of a collection of organized, qualitative data including positive feedback and common opinions that were gathered during the competence-oriented instructional strategy implementation”, there is no significant difference after the statistical analysis. From the instruction and evaluation process results, the students of both the groups have worse performance in alphabet and graphics reading. There is no significant difference between the two groups who were not given a specific instruction. In terms of the skill component, the students in the experimental group were supposed to be able to appropriately apply the situated content to increase their creativity, professional skills, and practical capabilities to apply them to circuit design, application, and maintenance. In terms of the attitude component, the students in the experimental group focused more on their team expression and sharing during the instruction process, and therefore, those students who performed better were more likely to integrate with their classmates and express their opinions during the instruction process about the knowledge that they learned. The results in this study are consistent with those of many research studies. The study result from Huang (2018) showed that the implementation of competence-oriented instructional strategies help the students’ to cultivate creative thinking and problem-solving capabilities and also their communication ability [48]. Hsu (2018) indicated that the students’ learning effectiveness in competence-oriented instruction can be evaluated from the learners’ situated context in their daily lives [49].

#### 5.1.5. Evaluation of Qualitative Data about Competence-Oriented Instructional Strategies

In this study, the competence-oriented instruction activities improved the students’ knowledge, skills, and attitude, and at the same time, the integrated situated context of daily life and the current situation of industries allowed them to develop overall contextual instruction methods. Additionally, the instruction activities were appropriately adjusted according to the students’ learning condition during the instruction experiment period. The results of Table 21 (a statistical table of a collection of organized, qualitative data including positive feedback and common opinions that were gathered during the competence-oriented instructional strategy implementation) show that the students had a positive attitude toward the competence-oriented instructional strategies, in which the top three items that the lesson-observing teachers and students focused on were: “situational connection and expression”, “reading ability”, and “integrate interdisciplinary/cross-region and issues into subjects”. The following text about these three parts were taken from the lesson-observing teachers and students.

In terms of “situational connection and expression”, from the comprehensive evaluation result of the students’ performance in each stage, the students in the experimental group had a performed better in terms of the skill component than the students in the control group did (such as the examples of circuit switch position, the precaution about equipment maintenance, and the electricity utilization environment evaluation) because of the guidance from the instructors during the instruction about the observation of electricity utilization and the investigation of electricity utilization demand.

In terms of “reading ability”, there is no significant difference in the first learning effectiveness evaluation between the two groups in the condition of the students receiving no instruction during the graphics reading and information extraction tasks before the instruction experiment. Nevertheless, there is significant difference in the second evaluation result after adjusting the instruction activities in which the students in the experimental group performed better in the alphabet description, information extraction, and graphics reading tasks.

In terms of “integrating interdisciplinary/cross-region methods and issues into subjects”, aside from those that are mainly based on a specific topic, this study uses interdisciplinary/cross-region methods and issues, and it appropriately adjusts the curriculum topics for these. For example, to understand common household lamps, we must integrate different sources of optical knowledge in a natural setting, which requires us to conduct a further investigation of the impact of lamp’s ray casting and also its extended position in order to figure out the electricity utilization and energy consumption condition of different lamps.

As stated above, the results of this study are consistent with those of many related research studies. If we take the research from Hung, Tseng, and Wu (2018), for example, their research indicated that there is no fixed model, and a roll correction is needed according to the students’ learning performance [50]. Weng (2017) also indicated that it works on the cultivation of the students’ competency capabilities by panning the experimental, practical, and discussion instruction activities during the curriculum instruction [51].

## 6. Conclusions

This study came up with two results, which are presented as follows:

Competence-oriented instructional strategies implementation has a positive influence on students’ overall learning effectiveness compared with that of the traditional didactic instructional strategies. After conducting a further investigation of the learning effectiveness on the knowledge, skill, and attitude components, there was a significant difference in the skill and attitude components, in which the experimental group students’ learning effectiveness was better than that of the control group students. Even though there is no significant difference in terms of the knowledge component, the experimental group students obtained better average scores than the control group students did.

The implementation of the instructional activities in competence-oriented instructional strategies should be modified and adjusted based on the students’ learning situation, and at the same time, the reading ability should be emphasized. Through the triangulation of the qualitative data from the three observers and the roll correction of the instructional activities by instructors according to the qualitative data, it was found that there is a significant difference in the comparison of the students’ periodic learning effectiveness scores. In the summary of the research’s quantitative and qualitative data, the instructors should not only focus on planning and designing instructional activities, rather, the students’ reading competency is also a key point when they are implementing the competence-oriented instructions. There will not be a significant difference in the students’ learning performance in terms of the knowledge component in both the groups without pre-instruction and guidance in the reading part, before the instructional activities. Later, after the adjustment of instructional activities, which appropriately helped the students with their reading ability according to the instruction feedback, we found a significant difference in the second evaluation performance of the students.

In conclusion, the results of this study indicate that competence-oriented instructional strategies are useful and effective methods. Nevertheless, there are still two research limitations, which are stated below that can be used for future related research, as references. First of all, the competence-oriented instruction strategy has just started, so this research is limited to the field of the professional practice of working with indoor wiring and the integration of electronic circuit practice courses. We must make good use of internet multimedia to build the real-life-situated context that meets the instruction requirement. The instruction curriculum topic is “Basic Electricity Practice”, in which the real-life-situated context is not included as much as it is in other subjects, and there is also a limitation in the industry application expression. To help the students to pick it up quickly, combining internet multimedia and offering the students clear methods and implied meaning in industries and their daily lives during the curriculum instruction are good and effective practices. Therefore, it is hoped that this teaching method can be extended to other disciplines or related fields using the results of case studies. Yu (2021) indicated that in order to integrate the curriculum with real lives, daily practice is required [9]. The instructors should try to help the students to find ways to generalize their knowledge and skills to solve the problems and to meet the industrial requirement. Secondly, the students’ performance in information extraction and graphics application is based on their reading ability. It is suggested that the instructors should help the students to become more familiar with information extraction skills and graphics reading before the instruction and by questioning them during the instruction activities to guide the students to investigate and define the problems, and to turn this form of thinking into a habit, and further, to deepen the students’ reading capabilities. Thirdly, in terms of “integrating interdisciplinary/cross-region methods and issues into subjects”, we advise that researchers do not limit themselves to a single topic, as interdisciplinary and multiple issues and topics can be adjusted according to the curriculum, which makes it easier for instructors to use and at the same to reach the goal of achieving “half the work; twice the effect” in terms of the students’ learning performance. In addition, this study mainly relies on quantitative analysis, which is supplemented by qualitative feedback and data induction. The suggestion for future related research is to extend the research time and schedule in order to include multiple ways of adding students’ learning process files, choosing interdisciplinary/cross-region lesson-observing teachers, using classroom recording as a supporting material, and so forth, in order to obtain a complete picture of the learners in the implementation of competence-oriented instructional strategies. Lin and Chen (2019) indicated that it is necessary to not only ask the students to show how to answer with the knowledge and skills that they have learned. Therefore, the students’ performance evaluation usually equals to the combination of the students’ performance process and products [41]. As the result of the COVID-19 pandemic and the following governmental epidemic prevention regulations, distance instruction implementation has become a big challenging for practical subjects, as well as students’ learning effectiveness in skills-based senior high schools. Different from the prior instruction that focused on the knowledge content, competence-oriented instructional strategies emphasize designing questions that are based on the real-life-situated contexts to guide the students to solve the problems that they encounter in their daily lives by applying the knowledge that they have learned. As a consequence, it worth delving into and investigating the methods of how to integrate and apply competence-oriented instructions to the distance instruction implementations in skills-based senior high schools. To adapt to and meet the requirement in the new generation of digitally distanced instruction, we hope that the results in this study will effectively provide the instructors in skills-based senior high schools more understanding of instructional strategies.

## Figures and Tables

**Table 1:** Instruction experimental design.

Instruction Period	Grouping Method	Pretest	Experimental Treatment	Posttest
Experimental group	Heterogeneous grouping	O_1_	X_1_	O_2_
Control group	Heterogeneous grouping	O_3_	X_2_	O_4_

X_1_ Competence-oriented instructional strategies. X_2_ Traditional didactic instructional strategies.

**Table 2:** Evaluation rubric scale developed in this research.

	Level	Excellent 100–81	Very Good 80–61	Good 60–41	Fair 40–21	Poor 20–0
Performance						
**Knowledge (34%)**	Industrial safety, hygienic knowledge and power utilization	Be excellent in industrial safety, hygienic knowledge and power utilization	Be very good in industrial safety, hygienic knowledge and power utilization	Be good in industrial safety, hygienic knowledge and power utilization	Be fair in industrial safety, hygienic knowledge and power utilization	Be poor in industrial safety, hygienic knowledge and power utilization
	Circuit design, maintenance and problem definition	Be excellent in circuit design, maintenance and problem definition	Be very good in circuit design, maintenance and problem definition	Be good in circuit design, maintenance and problem definition	Be fair in circuit design, maintenance and problem definition	Be poor in circuit design, maintenance and problem definition
	Application of tools and instruments	Be excellent in application of tools and instruments	Be very good in application of tools and instruments	Be good in application of tools and instruments	Be fair in application of tools and instruments	Be poor in application of tools and instruments
**Skills (33%)**	Tools assembly and position planning	Be excellent in tools assembly and position planning (meet the situational requirements: more than 4 items)	Be very good in tools assembly and position planning (meet the situational requirements: 3 items)	Be good in tools assembly and position planning (meet the situational requirements: 2 items)	Be fair in tools assembly and position planning (meet the situational requirements: 1 item)	Be poor in tools assembly and position planning (fail to meet the situational requirements: 0 items)
	Tools assembly	Be able to complete the circuit planning and assembly before the deadline and comply with the rule requirements of electrical code	Be able to complete the circuit planning and assembly on the deadline and comply with the rule requirements of electrical code	Be able to complete the circuit planning and assembly after the deadline and comply with the rule requirements of electrical code	Be able to complete part of the circuit planning and assembly after the deadline and comply with the rule requirements of electrical code	Fail to complete the circuit planning and assembly after the deadline
	Operation after tools assembly	Be able to complete the tools assembly operation and design expression before the deadline	Be able to complete the tools assembly operation and design expression on the deadline	Be able to complete the tools assembly operation and design expression after the deadline	Be able to complete part of the tools assembly operation and design expression after the deadline	Fail to complete the c operation and design expression after the deadline
**Attitude (33%)**	Tools using and materials application performance during practical process	Circuit measurement, wire gauge and colors choice, and delamination of electric wire insulating cover inappropriate (0–3 items)	Circuit measurement, wire gauge and colors choice, and delamination of electric wire insulating cover inappropriate (3–5 items)	Circuit measurement, wire gauge and colors choice, and delamination of electric wire insulating cover inappropriate (6–8 items)	Circuit measurement, wire gauge and colors choice, and delamination of electric wire insulating cover inappropriate (9–11 items)	Circuit measurement, wire gauge and colors choice, and delamination of electric wire insulating cover inappropriate (include and over 12 items)
	Group sharing during practical process and performance of problem-solving capability	Be able to instruct most of the classmates who are far behind (4 persons)	Be able to instruct most of the classmates who are far behind (3-2 persons)	Be able to instruct most of the classmates who are far behind (1 person)	Be not able to instruct most of the classmates who are far behind	Need other classmates’ support to complete the practical process
	Tool maintenance and site preparation performance after practical process	Be excellent in tool maintenance and site preparation after practical process wire or element left: 0–3 items	Be very good in tool maintenance and site preparation after practical process wire or element left: 4–6 items	Be good in tool maintenance and site preparation after practical process wire or element left: 7–9 items	Be fair in tool maintenance and site preparation after practical process wire or element left: 10–12 items	Be poor in tool maintenance and site preparation after practical process

**Table 3:** Recapitulation sheet of students’ prior knowledge scores.

Item	Group	Number of Students	Average Value	Standard Deviation	*t* Value
Academic performance in electrical engineering practice	Control group	33	72.18	9.426	0.018
	Experimental group	35	72.14	7.923	

**Table 4:** Recapitulation sheet of students’ prior knowledge scores in knowledge, skills, and attitude.

Item	Group	Number of Students	Average Value	Standard Deviation	*t* Value
Knowledge	Control group	33	28.88	3.698	−2.351 *
	Experimental group	35	30.66	2.449	
Skills	Control group	33	23.91	3.156	2.610 *
	Experimental group	35	22.06	2.656	
Attitude	Control group	33	19.39	2.806	−0.043
	Experimental group	35	19.43	3.720	

Note: * *p* < 0.05.

**Table 5:** Recapitulation sheet of basic electricity practical learning effectiveness scores.

Item	Group	Number of Students	Average Value	Standard Deviation	*t* Value
The first assessment of learning effectiveness	Control group	33	66.97	10.120	−0.472
	Experimental group	35	68.17	10.888	
The second assessment of learning effectiveness	Control group	33	69.55	8.779	−2.928 **
	Experimental group	35	75.63	8.328	

Note: ** *p* < 0.01.

**Table 6:** Recapitulation sheet of basic electricity practical learning effectiveness scores in terms of knowledge.

Item	Group	Number of Students	Average Value	Standard Deviation	*t* Value
The first assessment of learning effectiveness	Control group	33	25.55	3.800	−0.323
	Experimental group	35	25.86	4.153	
The second assessment of learning effectiveness	Control group	33	23.27	2.908	−2.823 **
	Experimental group	35	25.20	2.709	

Note: ** *p* < 0.01.

**Table 7:** Recapitulation sheet of basic electricity practical learning effectiveness scores in terms of skill.

Item	Group	Number of Students	Average Value	Standard Deviation	*t* Value
The first assessment of learning effectiveness	Control group	33	23.00	3.666	−0.649
	Experimental group	35	23.60	3.957	
The second assessment of learning effectiveness	Control group	33	24.00	3.597	−3.131 **
	Experimental group	35	25.20	2.709	

Note: ** *p* < 0.01.

**Table 8:** Recapitulation sheet of basic electricity practical learning effectiveness scores in terms of attitude.

Item	Group	Number of Students	Average Value	Standard Deviation	*t* Value
The first assessment of learning effectiveness	Control group	33	18.42	2.693	−0.434
	Experimental group	35	18.71	2.824	
The second assessment of learning effectiveness	Control group	33	22.27	2.601	−2.536 *
	Experimental group	35	23.91	2.737	

Note: * *p* < 0.05.

**Table 9:** Homogeneity of regression coefficients within groups in overall learning effectiveness data.

Resource	Type III Sum of Squares	*df*	Mean Square	*F*	*p*
Different instructional strategies * academic performance in electrical engineering practice	68.021	1	68.021	2.192	0.144

**Table 10:** Recapitulation sheet of analysis of covariance in basic electricity practical overall learning effectiveness.

Source of Variation	SS	*df*	MS	*F*	*p*
Academic performance in electrical engineering practice	2447.118	1	2447.118	77.446 ***	0.000
Instructional strategies	228.740	1	228.740	7.239 **	0.009
Deviation	2053.843	65	31.598		
Total	339187.500	68			

Note: *** *p* < 0.001; ** *p* < 0.01.

**Table 11:** Recapitulation sheet of statistics in overall learning effectiveness of basic electricity practical.

Group	Number of Students	Average Value	Standard Deviation	Average Value after Adjustment
Control group	33	68.26	8.23	68.24
Experimental group	35	71.90	8.29	71.91

**Table 12:** Homogeneity of regression coefficients within groups in overall learning effectiveness-knowledge component.

Resource	Type III Sum of Squares	*df*	Mean Square	*F*	*p*
Different instructional strategies * academic performance in electrical engineering practice-knowledge component	13.474	1	13.474	2.725	0.104

**Table 13:** Recapitulation sheet of analysis of covariance in basic electricity practical overall learning effectiveness.

Source of Variation	SS	*df*	MS	*F*	*p*
Academic performance in electrical engineering practice-knowledge component	243.231	1	243.231	47.914 ***	0.000
Instructional strategies	0.009	1	0.009	0.002	0.966
Deviation	329.968	65	5.076		
Total	43,044.500	68			

Note: *** *p* < 0.001.

**Table 14:** Recapitulation sheet of statistics in overall learning effectiveness of basic electricity practical -knowledge component.

Group	Number of Students	Average Value	Standard Deviation	Average Value after Adjustment
Control group	33	24.41	2.94	24.97
Experimental group	35	25.53	2.95	25.00

**Table 15:** Homogeneity test of regression coefficient in overall learning effectiveness in terms of the skill component.

Resource	Type III Sum of Squares	*df*	Mean Square	*F*	*p*
Different instructional strategies * academic performance in electrical engineering practice-skill component	1.723	1	1.723	0.366	0.547

**Table 16:** Recapitulation sheet of analysis of covariance in basic electricity practical overall learning effectiveness in terms of the skill component.

Source of Variation	SS	*df*	MS	*F*	*p*
Academic performance in electrical engineering practice-skill component	321.652	1	321.652	69.062 ***	0.000
Instructional strategies	134.986	1	134.986	28.983 ***	0.000
Deviation	302.734	65	4.657		
Total	40,823.750	68			

Note: *** *p* < 0.001.

**Table 17:** Recapitulation sheet of statistics in overall learning effectiveness of basic electricity practical in terms of the skill component.

Group	Number of Students	Average Value	Standard Deviation	Average Value after Adjustment
Control group	33	23.50	3.19	22.78
Experimental group	35	25.06	2.96	25.74

**Table 18:** Homogeneity test of regression coefficient in overall learning effectiveness in terms of the attitude component.

Resource	Type III Sum of Squares	*df*	Mean Square	*F*	*p*
Different instructional strategies * academic performance in electrical engineering practice-attitude component	0.023	1	0.023	0.008	0.931

**Table 19:** Recapitulation sheet of analysis of covariance in basic electricity practical overall learning effectiveness in terms of the attitude component.

Source of Variation	SS	*df*	MS	*F*	*p*
Academic performance in electrical engineering practice in terms of the attitude component	165.474	1	165.474	56.517 ***	0.000
Instructional strategies	15.304	1	15.304	5.227 **	0.026
Deviation	190.311	65	2.928		
Total	29,920.250	68			

Note: *** *p* < 0.001; ** *p* < 0.01.

**Table 20:** Recapitulation sheet of statistics in overall learning effectiveness of basic electricity practical in terms of the attitude component.

Group	Number of Students	Average Value	Standard Deviation	Average Value after Adjustment
Control group	33	20.35	2.20	20.36
Experimental group	35	21.31	2.43	21.31

**Table 21:** Statistical table of a collection of organized, qualitative data including positive feedback and common opinions that were gathered during competence-oriented instructional strategy implementation.

Content	Teacher (A)	Teacher (B)	Student (C)	Times
Situational connection and expression—positive	7	6	7	20
Reading ability	6	6	4	16
Integrate interdisciplinary/cross-region and issues into subjects	4	6	4	14
Problem definition and solving capabilities	4	3	2	9
Extend the practical training hours	3	2	3	8
Help classmates solve the problems	0	0	6	6
Situational connection and expression—negative			4	4
Situational expression, irrelevant to curriculum			2	2

**Table 22:** Statistical table of a collection of organized, qualitative data including lots of feedback and common opinions that were gathered during competence-oriented instructional strategy implementation.

Item	A Teacher			B Teacher			Experimental Group Students		
	Agree	Neutral	Disagree	Agree	Neutral	Disagree	Agree	Neutral	Disagree
Situational connection and expression	7/24	1/24	0/24	6/24	2/24	0/24	7/24	1/24	4/24
Reading ability	0/24	2/24	6/24	0/24	2/24	6/24	0/24	4/24	4/24
Integrate interdisciplinary/cross-region and issues into subjects	4/24	4/24	0/24	6/24	2/24	0/24	4/24	4/24	0/24
Problem definition and solving capabilities	4/24	4/24	0/24	3/24	5/24	0/24	2/24	6/24	0/24
Extend the practical training hours	0/24	5/24	3/24	0/24	6/24	2/24	0/24	5/24	3/24
Help classmates solve the problems	0/24	8/24	0/24	0/24	8/24	0/24	6/24	2/24	0/24

**Table 23:** Statistical table of a collection of organized, qualitative data including observational coefficients and common opinions that were gathered during competence-oriented instructional strategy implementation.

Item	Total Amount of 3 Observers’ Opinions					
	Coefficient of Agreement		Coefficient of Neutralization		Coefficient of Disagreement	
Situational connection and expression	20/24	0.83	4/24	0.17	4/24	0.17
reading ability	0/24	0.00	8/24	0.33	16/24	0.67
Integrate interdisciplinary/cross-region and issues into subjects	14/24	0.58	10/24	0.42	0/24	0.00
Problem definition and solving capabilities	9/24	0.38	15/24	0.63	0/24	0.00
Extend the practical training hours	0/24	0.00	16/24	0.67	8/24	0.33
Help classmates solve the problems	6/24	0.25	18/24	0.75	0/24	0.00

## Data Availability

Not applicable.
